# Exploring Relationships between the Density of Charged Tracts within Disordered Regions and Phase Separation

**Published:** 2020

**Authors:** Ramiz Somjee, Diana M. Mitrea, Richard W. Kriwacki

**Affiliations:** Department of Structural Biology, St. Jude Children’s Research Hospital, 262 Danny Thomas Place Memphis, Tennessee 38105, USA; Department of Chemistry, Rhodes College, 2000 North Parkway Memphis, Tennessee 38112, USA; Department of Structural Biology, St. Jude Children’s Research Hospital, 262 Danny Thomas Place Memphis, Tennessee 38105, USA; Department of Structural Biology, St. Jude Children’s Research Hospital, 262 Danny Thomas Place Memphis, Tennessee 38105, USA; Department of Microbiology, Immunology, and Biochemistry, University of Tennessee Health Sciences Center, 910 Madison Avenue, Memphis, Tennessee 38163, USA

**Keywords:** Intrinsically disordered proteins, biomolecular condensates, phase separation, charge patterning

## Abstract

Biomolecular condensates form through a process termed phase separation and play diverse roles throughout the cell. Proteins that undergo phase separation often have disordered regions that can engage in weak, multivalent interactions; however, our understanding of the sequence grammar that defines which proteins phase separate is far from complete. Here, we show that proteins that display a high density of charged tracts within intrinsically disordered regions are likely to be constituents of electrostatically organized biomolecular condensates. We scored the human proteome using an algorithm termed ABTdensity that quantifies the density of charged tracts and observed that proteins with more charged tracts are enriched in particular Gene Ontology annotations and, based upon analysis of interaction networks, cluster into distinct biomolecular condensates. These results suggest that electrostatically-driven, multivalent interactions involving charged tracts within disordered regions serve to organize certain biomolecular condensates through phase separation.

## 1. Introduction

Biological liquid-liquid phase separation is a process through which biomolecules demix from their cellular environment, creating dense liquid- or gel-like condensates.^1,2^ Analogous to how oil forms droplets in water, phase separation results in intracellular biomolecular condensates, often containing myriad protein and nucleic acid components, with unique chemical properties. One role for these compositionally complex condensates is to create microenvironments that facilitate and organize the biochemical reactions needed to sustain life.^1^ For this reason, many biomolecular condensates are also referred to as “membraneless organelles.” Different condensates can serve different purposes: stress granules, for example, are cytoplasmic bodies that sequester mRNA during cellular stress; nuclear speckles serve as RNA processing centers; and nucleoli mediate ribosome biogenesis and cellular stress sensing.^1^ While much is understood, there are many condensates whose functions is still incompletely defined.

Proteins undergo phase separation when self-interactions are energetically more favorable than interaction with solvent molecules. However, the formation of two separate phases (*e.g.,* solvent-rich light phase and protein-rich dense phase) reduces the entropy of the system. This decrease in entropy is counter-balanced by favorable enthalpic interactions in the two phase system.^2^ Phase separation is driven by weak and transient, multivalent interactions within the dense phase which enable each individual component to transiently interact with several other component molecules simultaneously. Multivalency gives rise to networks of intermolecular contacts that organize the dense phase of condensates. These networks of non-covalently inter-linked molecules within liquid-like condensates create microenvironments that mediate a wide range of cellular processes.^1^

**Figure 1.**
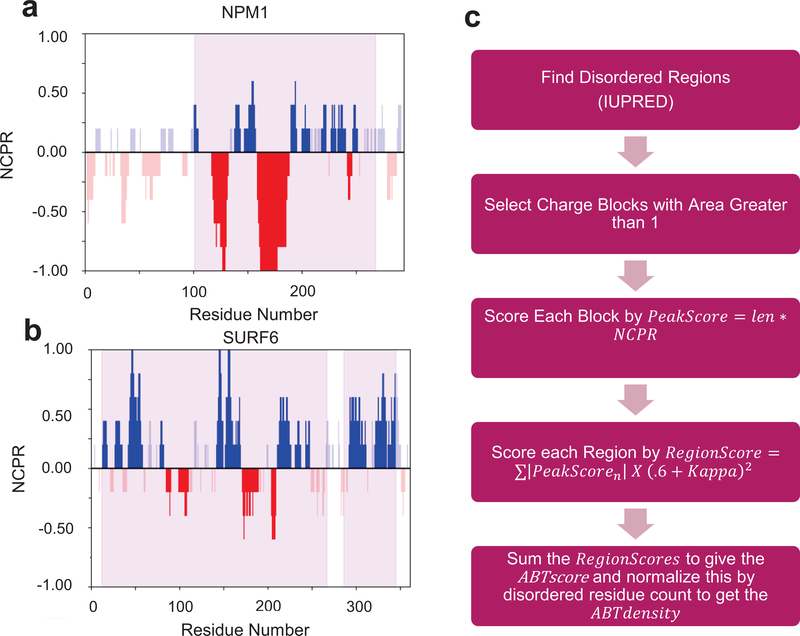
Net charge per residue plots of NPM1 (a) and another nuclear protein, SURF6 (b). Regions of predicted disorder highlighted in purple. Highlighted charged tracts have area greater than 1 Process diagram for the calculation of ABTdensity (c).

The multivalent interactions associated with phase separation can involve folded domains,^3^ residues within intrinsically disordered protein regions (IDRs),^1,4^ or a combination of the two types of interactions. Folded domains in proteins known to phase separate often bind to short linear motifs (SLiMs) within the IDRs of other proteins. Multivalent display of these folded domains and of the disordered motifs enables phase separation.^3^ In addition to participating in interactions, folded domains commonly mediate oligomerization, which enhances the multivalency of the protein’s other domains and IDRs.^5^ Interactions between IDRs can be the primary drivers of protein phase separation, or they can contribute to multifarious interactions between IDRs and folded domains that, in combination, form intermolecular networks that underlie phase separation.^1^ As noted above, the interactions mediated by IDRs can involve SLiM/folded domain interactions,^3^ but are also known to involve pi electron-containing^6^ and charged amino acids.^7^ Pi electron-containing amino acids (*e.g.,* tyrosine, phenylalanine, arginine, glutamine, and glutamine) experience pi-pi and pi-cation interactions and, if enriched within an IDR, can drive multivalent interactions and phase separation. In addition, electrostatic interactions between clustered blocks, or tracts, of oppositely charged amino acids (*e.g.,* arginine and lysine, and glutamic acid and aspartic acid) within IDRs promote phase separation (Figure 1a and 1b).^7^ Termed complex coacervation,^8^ this mechanism of phase separation can occur between tracts of oppositely charged residues in different biomolecules (termed heterotypic phase separation; *e.g.,* the polycationic C-terminal IDR of histone H1 and DNA^9^) or within the same polypeptide [termed homotypic phase separation; *e.g.,* acidic and basic tracts within the central IDR of Nucleophosmin (NPM1)^7^ ]. However, while the contributions of pi-pi and pi-cation interactions to the phase separation of proteins with IDRs have been extensively discussed,^6^ the contributions of electrostatic interactions between oppositely charged tracts of amino acids have not been systematically evaluated. Experimental studies with charged residue scramble mutants of Ddx4 showed that the mere presence of charged residues is not sufficient to drive phase separation,^10^ and theoretical studies confirmed that rearranging Ddx4’s charged residues so that they are no longer in contiguous tracts disrupts electrostatic interactions driving phase separation.^11^ Accordingly, efforts to quantify the patterning of charged residues have introduced several sequence feature parameters such as the *kappa* parameter in the context of IDR ensembles ^12^ and the *sequence charge decoration* parameter in the context of phase separation.^13^ However, these parameters do not explicitly examine the occurrence of charged tracts, and their evaluation is from a physical rather than informatics perspective. Thus, using NPM1 as a model, we developed a novel sequence analysis algorithm, termed ABTscore, that quantifies the occurrence of tracts of acidic and basic residues in IDRs. Here we report the results of analysis of the human proteome using the ABTscore algorithm, Gene Ontology annotations, and protein interaction data. Ultimately, our results suggest that the density of charged tracts within IDRs can distinguish biomolecular condensates organized through electrostatic interactions. Proteins with a high density of charged tracts are enriched in particular gene ontology annotations, many of which already have ties to phase separation. Finally, an interaction network analysis revealed increased physical and genetic interactions amongst proteins with higher ABTdensity values. Clustering of these networks showed groups of proteins that appear to represent specific condensates. That these groups appear for proteins with a range of ABTvalues suggests the involvement of a client-scaffold model^14^ in the organization of electrostatically driven condensates.

## 2. Methods

### 2.1. ABTscore Algorithm

The ABTscore quantifies the presence of contiguous stretches of either acidic or basic residues, termed tracts, within IDRs. We focus on IDRs because the charged residues in a structured domain may or may not be available for intermolecular interaction and because IDRs have known roles in protein phase separation. We used IUPRED^15^ to calculate the per-residue disorder score, which was smoothed by calculating the rolling average over a window of seven residues in length. IDRs for further analysis were selected as those stretches where the smoothened disorder propensity was continuously greater than 0.45. However, IDRs within seven residues of each other were combined and analyzed together. Finally, IDRs that were shorter than 30 residues were excluded from further analysis. While these parameters were not rigorously optimized, they were selected to ensure that disordered regions in two proteins experimentally known to undergo phase separation, NPM1^5^ and NUP98,^16^ were identified by our algorithm to be disordered. Using these parameters, the occurrence of ~8 residues predicted to be structured would interrupt a predicted IDR.

Within each IDR, we calculated an average net-charge-per-residue (NCPR) value for each residue using a window five residues in length, a window length used previously in analyses of electrostatic interactions using Flory-Huggins theory.^12^ Using the NCPR values, we identified charged tracts as stretches of residues wherein the averaged NCPR value was positive or negative without interruption. Within each IDR, the sum of the area (area = number of residues × average NCPR) of charge blocks with an area greater than 1 was calculated. This sum was multiplied by *(0.6 + kappa)2*. The *kappa* parameter was used to quantify the extent of separation between acidic and basic residues within IDRs. When acidic and basic residues are well mixed (*e.g.,* DKDKDKDK), the *kappa* value is low; when the acidic and basic residues are separated (*e.g.,* DDDDKKKK), the *kappa* value is high.^12^ The rationale for this is that contiguous stretches of charged residues, as observed in NPM1, for example, are more likely to contribute to phase separation than stretches in which charged residues are dispersed. This procedure was repeated for each region of predicted disorder within a protein, and the ABTscore value was calculated as the sum of the score for each region. Finally, the ABTscore was normalized by the number of residues within a region of predicted disorder to calculate the ABTdensity (Figure 1c). The computational pipeline used to compute ABTscore and ABTdensity values for proteins was written in Python 3.7. Scripts are available upon request. All external modules except localcider^17^ are included in the Anaconda distribution, a standard library of python extensions (anaconda.com). IUPRED^15^ disorder information was computed locally using scripts reported in the publication.

### 2.2. Gene Ontology Enrichment Analysis

We determined ABTscore and ABTdensity values for all proteins in the non-redundant, reviewed human proteome [obtained from Uni-Prot (uniport.org)], accessed 7–11-2019). This analysis identified 10,946 proteins with regions of predicted disorder >30 residues, which were stratified according to ABTdensity values, as follows: Group 1 contained proteins with the top 5% of ABTdensity values; Group 2, those with scores ≤5% and >15%; Group 3, those with scores ≤15% and >30%; and Group 4, the remainder (Supplemental Data 1). Each protein Group was analyzed with respect to Gene Ontology^18,19^ process, function, and component enrichment using the PANTHER webtool.^20^ The results of enrichment analyses for proteins within each of the four Groups were obtained through comparison with the complete starting pool of disordered proteins (Groups 1–4). Fold enrichment, p-values and false discovery rates were reported by PANTHER^20^ according to the default settings. Data for each Gene Ontology term, enriched or not, was recorded. We considered terms with a 2-fold enrichment between the test Group and the complete disordered protein pool (Groups 1–4) at p ≤ 0.05 as enriched. To eliminate rare Gene Ontology terms, we prioritized annotations used more than 50 times in the disordered protein pool; other terms were excluded from analysis. If a large number of frequently used Gene Ontology terms were shown to be enriched in Group 1, the least indispensable terms according to the REVIGO web tool^21^ were selected for presentation in Figures. Input for REVIGO^21^ was the list of frequently used, enriched terms along with their fold enrichment. The full lists of terms and the enrichment results are found in Supplemental Data 2.

### 2.3. Interaction Network Analysis

We used the string-db webserver (string-db.org) to conduct an analysis of genetic and physical interactions on the proteins in each Group 1–3 (Supplemental Data 1). Group 4 was excluded because its size (n=7673) was larger than that allowed by the string-db webserver. Uniprot accession codes were used in the multiple protein mode to generate network graphs of each Group. We evaluated the network connectivity for each Group by comparing the number of observed interactions to the number of expected interactions within the same number of random proteins. We then used the built-in k-means algorithm to group proteins into 5 clusters. We evaluated the four smaller clusters with respect to the enriched Gene Ontology^18,19^ processes, function and component annotations. The fifth, largest cluster was excluded because it appeared to group proteins only on the basis of their exclusion from other clusters rather than on enhanced interactions. Fold enrichment compared to the human proteome and false discovery rates were calculated through the PATHER webserver.^20^ The full lists of terms and enrichment data are found in Supplemental Data 3. The fold enrichment was compared to the human proteome here instead of the proteins with IDRs because the interaction enrichment analysis was performed with the human proteome as the background. In the case of process and component analyses, we only analyzed terms with more than 50 usages in the human proteome. Terms with the highest-fold enrichment and highest usages within a cluster informed the identification of a cluster to a potential phase separated condensate. However, this identification was not possible in every case.

## 3. Results

**Figure 2.**
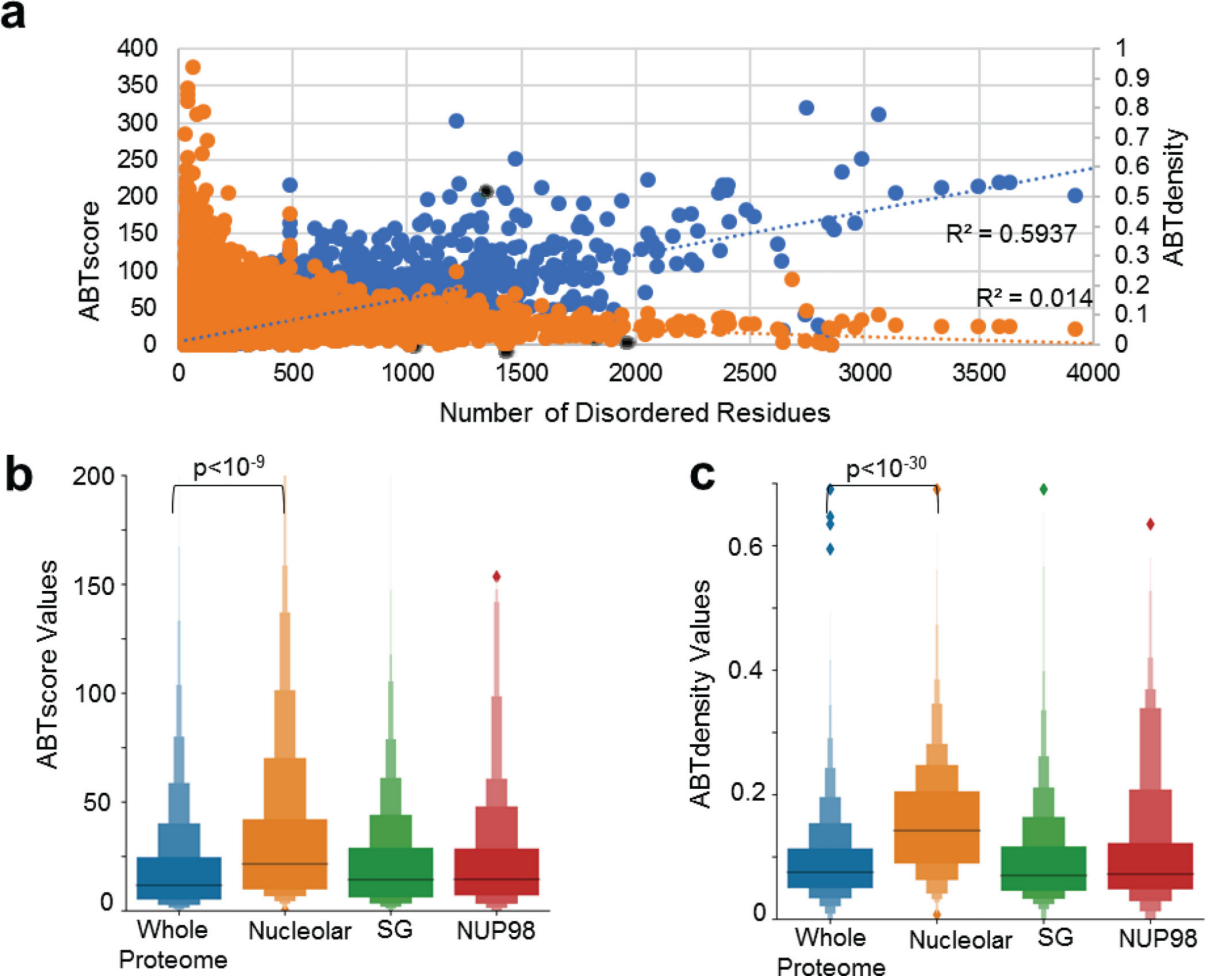
Scatter plot showing correlation between ABTscore values (blue data points) and ABTdensity values (orange data points), and the number of disordered residues in each protein with one or more disordered regions (a). Enhanced box plots showing ABTscore (b) and ABTdensity (c) distributions for the whole proteome, nucleolar proteome, stress granule (SG) proteome, and NUP98 interactors. P values are reported when a protein set’s mean is different from the whole proteome’s at a p<0.05.

To understand the prevalence and distribution of tracts of charged residues within IDRs, we calculated ABTscore and ABTdensity values for the human proteome. Approximately 45% (9,470 of 20,416 proteins) of the proteins analyzed lacked a disordered region >30 residues in length, consistent with past observations.^22^ Among those proteins with at least one region of predicted disorder >30 residues in length, most had low ABTscores as described below in Table 1. However, because the ABTscore is a cumulative value, the set of proteins with the largest ABTscore values displayed very long regions of disorder (Figure 2a). Thus, we reconsidered the proteome in terms of ABTscore values normalized by the number of residues within the disordered regions that were analyzed, giving the ABTdensity value. The ABTdensity values followed a similar distribution to the ABTscore where most proteins had low scores.

Next, we narrowed our focus from the entire proteome to proteins within specific phase separated bodies. We hypothesized that membraneless organelles formed through electrostatic interactions would be enriched in proteins with high ABTscore and ABTdensity values. Compared to the ABTscore value distribution for the entire proteome, nucleolar proteins^23^ exhibited an enrichment in ABTscore (median=22) (Figure 2a) and ABTdensity values (median=0.14) (Figure 2b). However, proteins from other bodies known to be formed by phase separation driven by hydrophobic interactions, such as stress granules,^24^ exhibited a slight enrichment of ABTscore (median=14, p = 0.06) but not ABTdensity (median=0.07, p=0.25) values (Figure 2a and 2b). Similarly, proteins that interact with Nucleoporin 98 (NUP98) [interactome from BioGRID (thebiogrid.org)], accessed 7–12-2019), a component of the phase separated permeability barrier in the nuclear pore,^25^ may have been slightly enriched in their ABTscore (median=15, p=0.16) but not their ABTdensity (median=0.07, p=0.09) values (Figure 2b and 2c). NUP98 and other components of the nuclear pore’s permeability barrier condense through hydrophobic interactions driven by an FG-repeat-rich IDRs.^25^ The nucleolus, on the other hand, is the center for production of ribosomal RNA (rRNA) and, through phase separation with NPM1 (Figure 1a) and other proteins displaying tracts of charged residues (Figure 1b), ribosomal proteins (rProteins) are sequestered within the nucleolus for assembly with rRNA to form ribosomal subunits. The ribosomal components, rRNA and rProteins, are highly charged and are present at high density within the nucleolus. The enrichment of tracts of charged residues in other, non-ribosomal nucleolar proteins may afford electrostatic compatibility to the ribosomal components and promote formation of the nucleolus through liquid-liquid phase separation.

**Figure 3.**
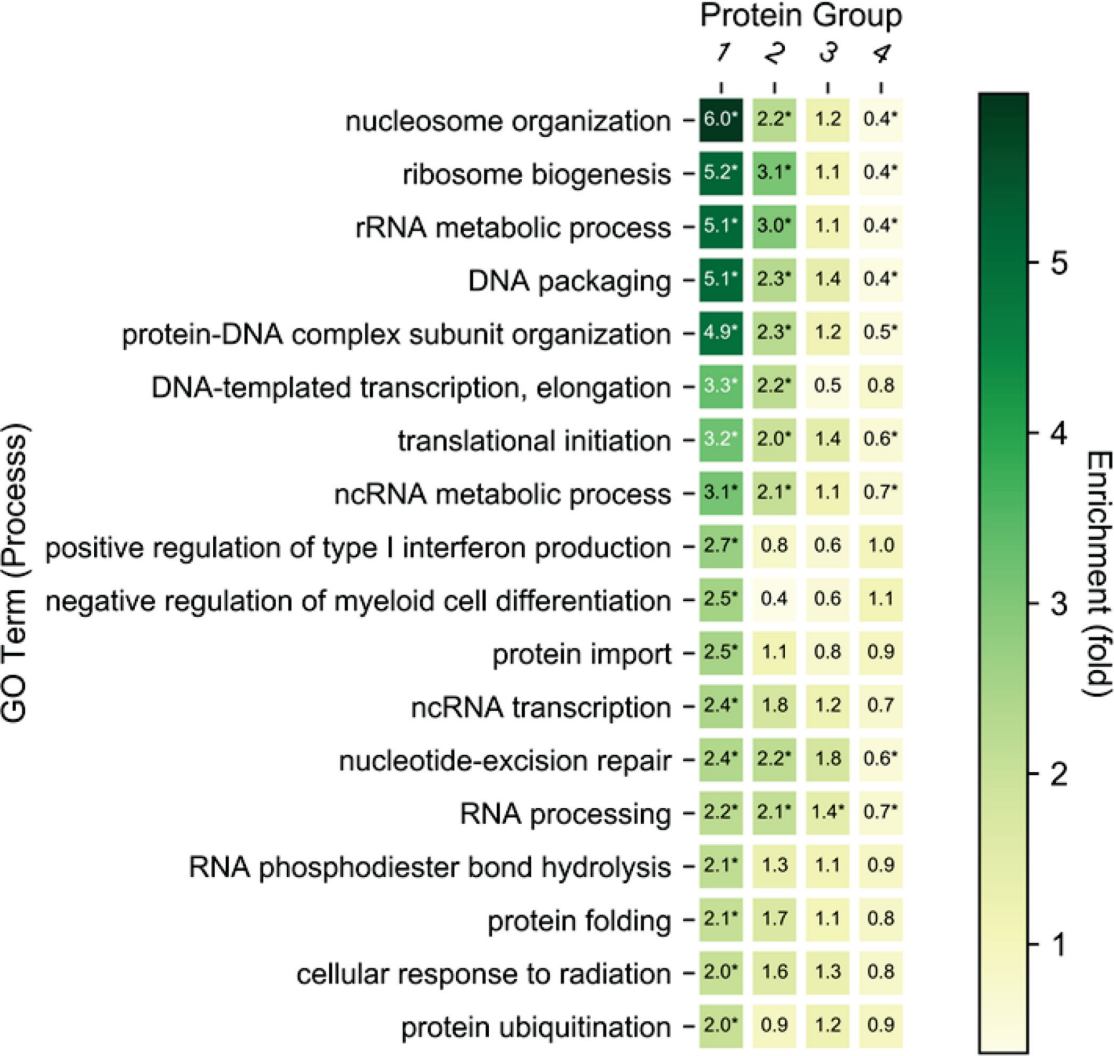
Heatmap of the Gene Ontology enrichment analysis for processes annotations of proteins in Groups 1 through 4. Asterisks indicate significance at p≤0.05.

We hypothesized that, if the ABTdensity value is an indicator of electrostatically driven phase separation, proteins with high ABTdensity should be enriched for particular functions because they would be localized within similar types of condensates. To test this hypothesis, we performed a Gene Ontology^18,19^ enrichment analysis^20^ and found that within the top 5% of proteins ranked by their ABTdensity value (Group 1), 176 process annotations are enriched more than two-fold with p ≤ 0.05 (Supplemental Data 2). Of these, 40 were frequently used terms. Many of the enriched terms relate to ribosome biogenesis, RNA processing, DNA organization, transcription, and its regulation (Figure 3). Enrichment for many terms is proportional to ABTdensity. For example, proteins in Group 1 are 5.2-fold enriched in ribosome biogenesis annotations. Proteins in Group 2 exhibited a 3.1-fold enrichment and those in Group 4 were deficient in ribosome biogenesis annotations (Figure 3). Enrichment analysis in terms of function and component annotations leads to 71 and 43 enriched terms, respectively, for proteins in Group 1 (Supplemental Data 2); 18 and 13, respectively, of these enriched annotations are frequently used terms. Many of the enriched functional terms are associated with RNA and nucleosome binding (Supplemental Figure 1a) while many enriched component terms relate to the nucleosome, RNA polymerase complex, and preribosome (Supplemental Figure 1b).

**Figure 4.**
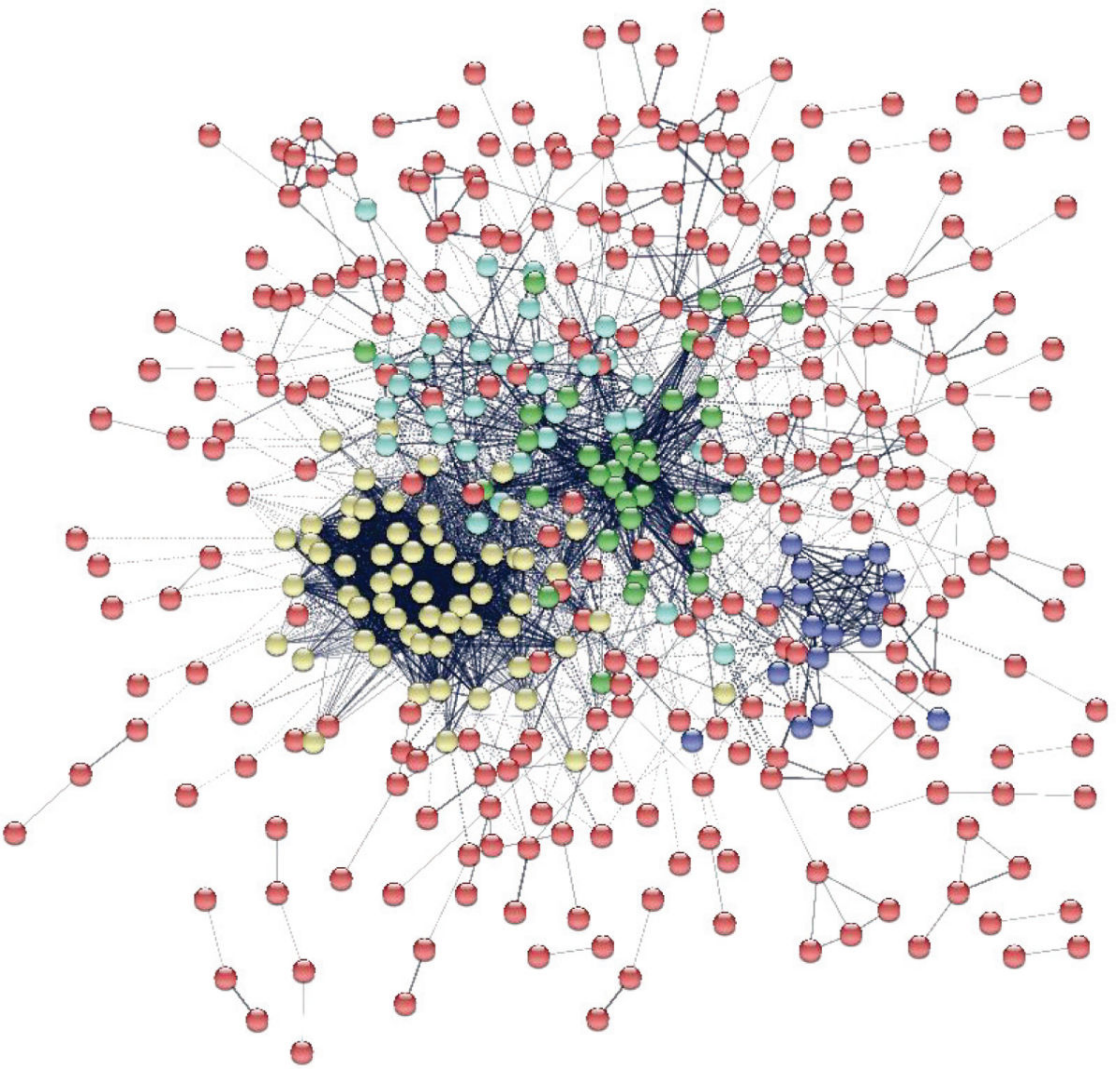
Interaction network for Group 1. Nodes represent proteins and edges represent physical or genetic interactions. Orphan proteins are not shown. Each color represents clusters described in Table 3. Yellow, nucleolar proteins; green, proteins associated with nucleosomes and heterochromatin; cyan, proteins associated with transcription bodies; and blue, proteins associated with protein degradation.

We additionally hypothesized that proteins with high ABTdensity values should have enriched physical and genetic interactions amongst themselves because they might function together within specific condensates. To test this idea, we generated interaction network graphs for Groups 1–3 where proteins are represented as nodes and interactions as edges (Figure 4, Supplemental Figure 2a and 2b). We found that proteins in each Group have enriched interactions as shown below in Table 2. Interestingly, the fold enrichment of interactions for each group is approximately proportional to mean ABTdensity value (Table 1).

Finally, we determined whether proteins associated with specific condensates or membraneless organelles could be identified within these networks by clustering proteins within each of the Groups. Based on the Gene Ontology terms for the clusters in Group 1 (Table 3), we propose that the 4 clusters (Figure 4) arise due to phase separation of proteins with high ABTdensity values within particular biomolecular condensates, including the nucleolus, nucleosomes or heterochromatin, transcription bodies, and protein degradation (Figure 4 and Table 3). A similar analysis of the clusters from Group 2 led to suggestions of the associated biomolecular condensates but these associations were more ambiguous than observed with Group 1. Results for Group 3 were similarly ambiguous (Supplemental Table 1 and Supplemental Figure 2). This trend that several clusters associated with proteins in Groups 1–3 appear to represent phase separated condensates suggests that a client-scaffold^14^ model organizes electrostatically driven condensates where the proteins with high ABTdensity values drive phase separation and others associate with their lesser charge tract features.

## 4. Discussion

IDRs contribute many of the weak, multivalent interactions needed to drive protein phase separation;^4^ however, the role of electrostatic interactions has not been broadly explored. Our results show that the density of charged tracts within IDRs correlates with phase separation and, combined with proteomic data, can distinguish distinct condensates within the human proteome. An important further implication is that electrostatic forces may be important in the phase separation of proteins associated with the processes and condensates described in Figure 3.

Using ABTdensity values to segregate the proteome and perform a Gene Ontology enrichment analysis revealed the enrichment of many annotations (Figure 3). While the fact that several annotations are enriched supports correlation between ABTdensity values and phase separation, many of the enriched annotations are already known to be associated with phase separation. The known roles of phase separation in the nucleolus,^26^ RNA processing,^27^ DNA organization,^28^ and transcription^29^ further support the conclusion that the Gene Ontology enrichments are due to phase separation and not some other mechanism dependent on the density of charged tracts. The enrichment of these specific terms also indicates that electrostatic interactions might be driving the formation of the condensates that organize these processes.

Additionally, that enrichment smoothly decreased across the four protein Groups (Figure 3) rather than being discontinuous suggests that there may not be a single cut-off value of the ABTdensity that indicates phase separation. Rather, proteins with the highest scores might serve as scaffolds that organize condensates while proteins with intermediate ABTdensity values associate as clients. Both clients and scaffolds are vital for condensate function, and the analysis of ABTdensity values may serve as a method to facilitate identification of clients where the known scaffolds already have high ABTdensity values. This client-scaffold model^14^ also explains why interaction fold enrichment was decreasing but still statistically enriched across Groups 1–3 (Table 2) and why clusters across Groups 1–3 can be recognized as biomolecular condensates, though with varying clarity (Table 3, Figure 4, Supplemental Figure 2 Supplemental Table 1).

We recognize that electrostatic forces are not the only contributing factor to the phase separation of IDRs within proteins. Studies showing that arginine to lysine mutations decrease phase separation propensity demonstrate that, even amongst charged residues, additional interactions, such as pi contacts, may be relevant to phase separation.^6^ Comparing the results of Gene Ontology enrichment as a function of ABTdensity (Figure 3) to a similar analysis conducted based upon analysis of pi-contact based phase separation (using PScore values)^6^ reveals some overlapping but many distinct terms. Both scores show an enrichment for chromatin annotations and terms related to RNA processing. However, high PScore proteins show an enrichment in cytoskeleton terms while proteins with high ABTscores are deficient in these terms. Likewise, enriched terms related to ribosome biogenesis and DNA organization in proteins with high ABTscores were not reported as enriched for proteins with high PScores. These overlaps and distinctions suggest that while some phase separated bodies depend on both electrostatic and pi contacts, many phase separated bodies have a dominating mechanism.

Fin ally, the ABTdensity value is a sequence-based parameter, but its correlation to phase separation has roots in the physical chemistry of polypeptide chains. Computational studies have shown that the distribution of charged residues within a peptide influences its conformational properties. When charges are well mixed (no tracts, low ABTdensity value), peptides have larger radii of gyration. As charged residues are segregated into tracts (high ABTscore), the peptides become more compact as a result of intramolecular, electrostatic interactions.^12^ Links between intra- and inter-molecular interactions suggest that a similar compaction should allow proteins with a high ABTdensity value to form condensates. But, because the ABTdensity does not account for a balance between positive and negative charge tracts, it may be more useful for identifying proteins likely to be involved with a biomolecular condensate rather than individual proteins that can homotypically phase separate *in vitro*. While this study directly shows that the density of charged tracts in a disordered protein region correlates with its function, the mechanistic relationship between this correlation and phase separation can only inferred from bioinformatic studies. Ultimately, computational methods, such as those that use course-grained approaches to simulate peptides,^13^ are needed to test our hypothesis that proteins with higher ABTdensity values have increased phase separation propensity. Experimental studies of interest include investigating whether proteins with high ABTdensity values actually partition into the biomolecular condensates predicted by the clustering analysis (Figure 4 and Table 3) in an ABTdensity dependent manner.

## Supplementary Material

Supplemental Figures and Table

Supplementary Data 1

Supplementary Data 2

Supplementary Data 3

## Figures and Tables

**Table 1.** Table showing the ABTscore and ABTdensity value ranges for different percent ranges of proteins. The designated groups and number of proteins (N) are based on ABTdensity.

Protein Percent Range	ABTscore Value Range	ABTdensity Value Range	Mean ABTdensity Value	N

Top 5% (Group 1)	64–650	0.21–0.94	0.29	537
≤5% to <15% (Group 2)	36–54	0.12–0.21	0.21	1094
≤15% to < 30% (Group 3)	22–36	0.10–0.14	0.14	1642
Remaining (Group 4)	0–22	0–0.10	0.06	7673

**Table 2.** Table showing the results of the interaction enrichment analysis for each Group 1–3. Enrichment P-value

Group	Observed Interactions	Expected Interactions	Interaction Fold Enrichment (O/E)	Enrichment P-value

1 (n=513)	2624	1289	2.04	<10^−16^
2 (n=1045)	7147	4700	1.52	<10^−16^
3 (n=1579)	10382	7021	1.22	<10^−16^

**Table 3.** Table showing Gene Ontology (GO) terms associated with proteins within each cluster from the interaction network of Group 1 (see Figure 4) and identification of the potential condensate each cluster represents. N is the number of proteins in each cluster.

Cluster	N	GO Process	GO Function	GO Component	Condensate

Yellow	60	rRNA processing, ribosome biogenesis	snoRNA binding, translation initiation factor activity, RNA helicase activity	Nucleolus, Cajal body	Nucleoli
Green	36	Histone, and chromatin binding, dimerization activity	Histone, and chromatin binding, dimerization activity	Nucleosome, heterochromatin, PML body,	Nucleosome and heterochromatin
Cyan	34	RNA polymerase activity, transcription initiation activity	RNA polymerase activity, transcription initiation activity	RNA polymerase complex	Transcription bodies
Blue	18	Ubiquitin, ubiquitin-like, protein transferase activity	Ubiquitin, ubiquitin-like, protein transferase activity	Ubiquitin ligase complex, transferase complex	Protein Degradation
