# Supplementary material for: Exploring Relationships between the Density of Charged Tracts within Disordered Regions and Phase Separation
Source: Pac Symp Biocomput. Author manuscript; Available in PMC 2020 Jan 2. (PMC6939312)
Supplement: Supplemental Figures and Table [file NIHMS1061152-supplement-Supplemental_Figures_and_Table.pdf]

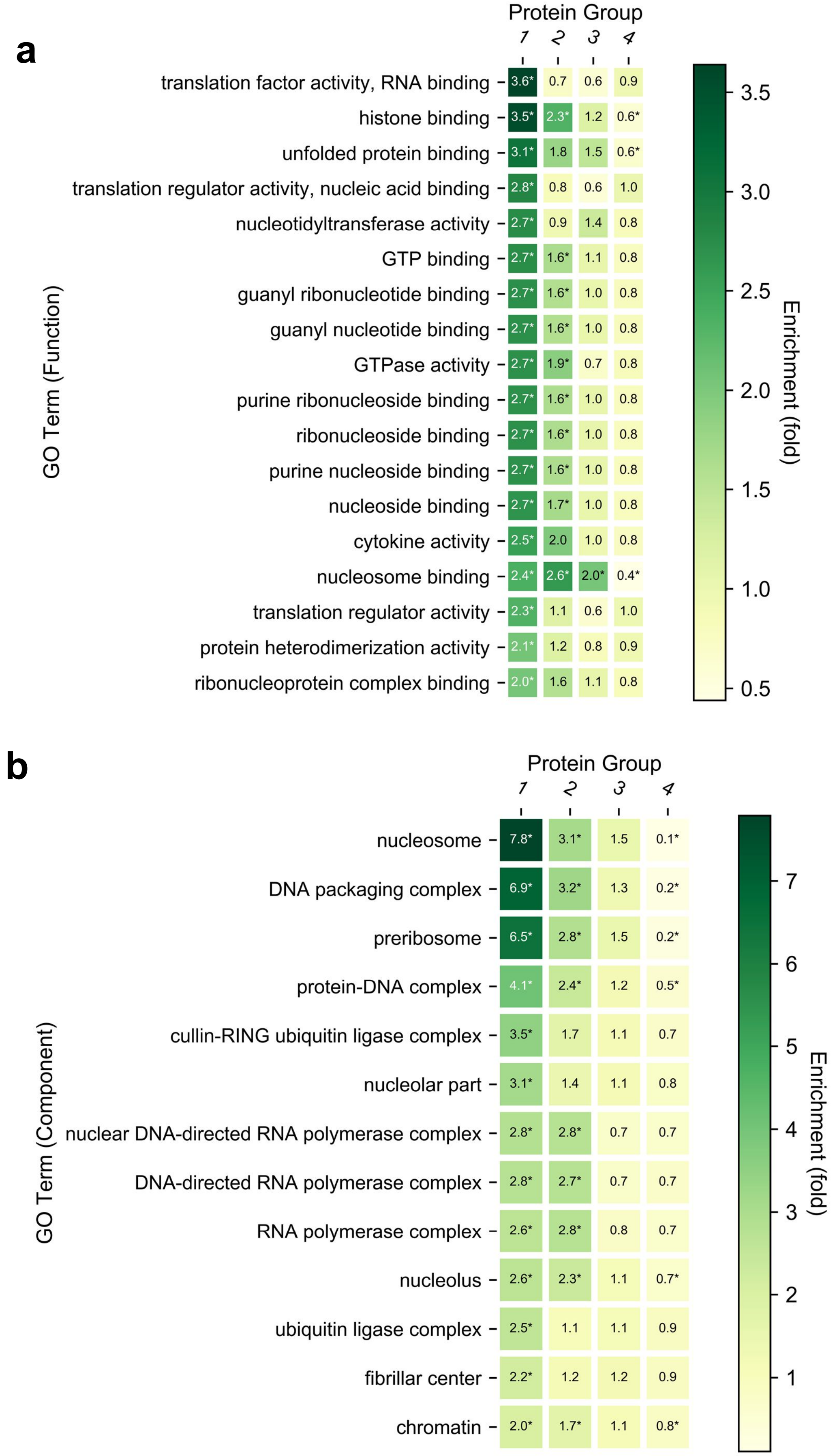

Supplemental Figure 1. Heatmaps of the Gene Ontology enrichment analysis for function (a) and component (b) annotations of proteins in Groups 1 through 4. Asterisks indicate significance at $p \leq 0.05$.

Supplemental Table 1. An analogue of Table 3 for Groups 2 and 3. Table showing the Gene Ontology (GO) terms associated with each cluster for interaction network analyses of Groups 2 and 3. Colors correlate with plots in Supplemental Figure 2a and 2b.

Group	Cluster	N	GO Process	GO Function	GO Component	Condensate
2	Yellow	76	Chromatin organization, histone modification	Methylated histone and nucleosome binding,	Heterochromatin, transcriptional repressor complex	Heterochromatin
2	Green	67	Ribosome biogenesis, rRNA processing	RNA binding, RNA helicase activity	Nucleolus, preribosome	Nucleolus
2	Cyan	64	mRNA processing, splicing, export	Structural constituent of ribosome, RNA binding	Ribosome, spliceosome, nuclear speckle	Nucleolus, nuclear speckle,
2	Blue	39	DNA repair and replication	Receptor binding	PML bodies, heterochromatin,	???
3	Yellow	122	Differentiation, morphogenesis	Various receptor binding	Apical or tight junction, membrane raft	???
3	Green	94	RNA splicing, chromatin silencing,	Helicase activity, pre-mRNA binding	Spliceosomal complex, nuclear speckle, transcriptional repressor complex	Nuclear speckle, RNA processing body
3	Cyan	62	Nucleosome and chromatin assembly,	Nucleosome and histone binding	Nucleosome, heterochromatin,	DNA organizing
3	Blue	55	Ribosome biogenesis, protein targeting to membrane or ER, rRNA processing	Structural component of ribosome, rRNA binding	Ribosome, spliceosome	Nucleolus, spliceosome

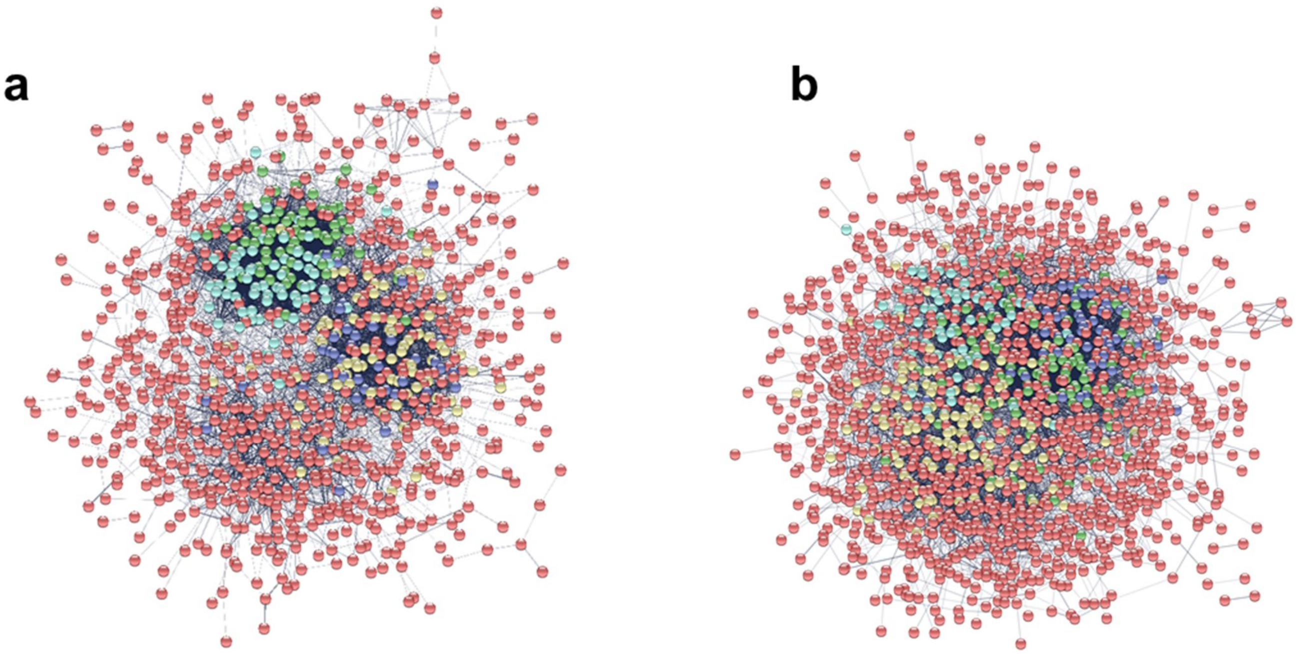

Supplemental Figure 2. Interaction network for Group 2 (a) and 3 (b). Nodes represent proteins and edges represent interactions. Orphan proteins are not shown. Each color represents clusters described in Supplemental Table 1.
